# Assessing exhibition swine as potential disseminators of infectious disease through the detection of five respiratory pathogens at agricultural exhibitions

**DOI:** 10.1186/s13567-019-0684-5

**Published:** 2019-09-18

**Authors:** Sarah E. Lauterbach, Sarah W. Nelson, Meghann E. Robinson, Josh N. Lorbach, Jacqueline M. Nolting, Andrew S. Bowman

**Affiliations:** 1 Department of Veterinary Preventive Medicine, The Ohio State University, 1920 Coffey Road, Columbus, OH 43210 USA; 2 Present Address: Health Science District, University of California Davis, 1 Garrod Drive, Davis, CA 95616 USA

## Abstract

Widespread geographic movement and extensive comingling of exhibition swine facilitates the spread and transmission of infectious pathogens. Nasal samples were collected from 2862 pigs at 102 exhibitions and tested for five pathogens. At least one pathogen was molecularly detected in pigs at 63 (61.8%) exhibitions. Influenza A virus was most prevalent and was detected in 498 (17.4%) samples. Influenza D virus was detected in two (0.07%) samples. More than one pathogen was detected in 165 (5.8%) samples. Influenza A virus remains a top threat to animal and human health, but other pathogens may be disseminated through the exhibition swine population.

## Introduction, methods, and results

Agricultural swine exhibitions showcase a variety of swine related educational programs, livestock auctions, and competitive shows for youth throughout the United States. Swine exhibitions are unique agricultural settings where large numbers of pigs from various geographic locales that would otherwise not come in contact with one another are co-housed and comingled for the length of the exhibition. Long exhibition durations (3–7 days), direct contact of swine, and relaxed biosecurity strategies have all been recognized as risk factors for the rapid transmission of infectious pathogens infecting swine during exhibitions [1, 2]. Influenza A virus (IAV) has been routinely detected in swine at agricultural exhibitions, where subclinical infection is common and can make recognition difficult [3–6]. Swine are important mixing vessels of IAV, where genetic reassortment between multiple IAV strains may produce novel, potentially more virulent strains [7]. The comingling of large numbers of IAV infected swine at exhibitions may increase the chance for the emergence of these reassortant viruses.

In addition to the extensive interaction of swine, exhibitions also create a unique human-animal interface, where exhibiting families and the general public are permitted to interact with swine. This unique swine-human interface facilitates zoonotic transmission of IAVs during swine exhibitions [8]. Continued detection of variant IAVs, IAVs infecting humans that normally circulate in swine, in individuals who have association with swine exhibitions has begun to heighten disease awareness and surveillance in exhibition swine [9, 10]. Combined with the ability of IAVs to reassort in swine, the increased swine-human interface of exhibitions creates opportunities for novel IAV strains to enter the human population, potentially leading to an influenza pandemic. Continued disease surveillance is vital to understanding the epidemiology of IAV and other pathogens infecting swine at agricultural exhibitions in order to protect animal and public health.

Commonly raised on small farms with varying management practices, the exhibition swine population is unlike commercial swine, which are managed in large herds under strict biosecurity protocols [11, 12]. Coupled with extensive movement over a large geographic area in order to attend multiple exhibitions, exhibition swine facilitate the widespread dissemination of infectious pathogens throughout the country [11, 13]. Therefore, exhibition swine represent a unique niche in the total swine population that may play an important role in the ecology and epidemiology of all swine infectious diseases. With continued detection of IAV and increasing awareness of disease in exhibition swine, the current study aims to identify pathogens in addition to IAV that may be circulating in this swine population. Other swine respiratory viruses such as porcine hemagglutinating encephalomyelitis virus (PHEV), porcine reproductive and respiratory syndrome virus (PRRSV), porcine parainfluenza virus 1 (PPIV1), and influenza D virus (IDV) have only briefly been described in exhibition swine and their prevalence and overall impact in exhibition swine remains unknown [12, 14–16]. With varying degrees of severity and impact on swine and human health, understanding the overall disease ecology and epidemiology of the exhibition swine population is vital to the development and implementation of disease mitigation strategies designed to protect animal and public health. Here, we describe the overall estimated prevalence of five infectious respiratory viruses in swine at agricultural exhibitions and assess the potential epidemiological role of exhibition swine.

In 2016, as part of an ongoing IAV surveillance program, 2862 pigs at 102 exhibitions across six states were sampled by nasal swab or nasal wipe [17, 18]. All samples underwent nucleic acid extraction and were tested individually for IAV using real-time reverse transcription polymerase chain reaction (rRT-PCR) as previously described [19]. Five microliters of extracted nucleic acid of no more than seven individual pigs from the same exhibition were pooled and screened for PPIV1, PHEV, and IDV using rRT-PCR as previously described [14, 16, 20] and for PRRSv using the VetMAX PRRSv reagents and manufacturer’s protocol (ThermoFisher Scientific, Waltham, MA, USA). Nucleic acid of samples within positive pools was subsequently tested individually using the same methods.

During field sample collection, exhibitions with ≥ 1 pig showing clinical signs consistent with influenza like illness (ILI) were recorded as having pigs with ILI [1]. Five of the exhibitions were excluded from statistical analysis due to lack of clinical sign data. Logistic regression (Stata special edition 14.2, College Station, TX, USA) was performed to determine any association between ILI noted at the exhibition and the detection of a pathogen in the pigs.

At least one pathogen was detected in the pigs at 63 (61.8%) of the 102 swine exhibitions tested. Influenza A virus was detected among the pigs at 37 (36.3%) and was detected at more exhibitions than any of the other viruses. In contrast, Influenza D virus was detected in the pigs at the fewest exhibitions; only pigs at two (2.0%) of the exhibitions tested positive for IDV (Table 1). More than one pathogen was detected among the pigs at 31 (30.4%) exhibitions; four pathogens were detected at three (2.9%), representing the highest number of pathogens detected among the pigs at any of the exhibitions. Exhibitions with pigs testing positive for at least one pathogen had 2.4 times the odds of having noted ILI compared to exhibitions where no pathogens were detected (*p* < 0.05). Detection of IAV in swine at exhibitions was the only pathogen with a significant positive association to noted ILI at exhibitions (OR = 1.3, *p* < 0.05). However, with fewer positives, this association may be hidden for the other pathogens.

**Table 1:** **Number of agricultural exhibitions with pigs testing positive for respiratory viruses by state**

State	Negative	IAV	PPIV1	PHEV	PRRSv	IDV
Ohio	20	13	15	12	6	1
Indiana	12	17	14	14	5	1
Michigan	8	5	1	0	1	0
West Virginia	1	0	0	0	0	0
Kentucky	0	1	1	1	0	0
Iowa	0	1	1	1	1	0
Total	41	37	32	28	13	2

Individual swine nasal samples from 102 agricultural exhibitions across six states were tested for five respiratory viruses using real-time reverse transcription polymerase chain reaction. The number of swine exhibitions with ≥ 1 pig positive for each pathogen are shown by state. Out of the 102 exhibitions tested, at least one pathogen was detected in the pigs at 63 (61.8%). The pigs at 31 (30.4%) exhibitions were positive for more than one virus.

IAV: influenza A virus, PPIV1: porcine parainfluenza virus 1, PHEV: porcine hemagglutinating encephalomyelitis, PRRSv: porcine reproductive and respiratory syndrome virus, IDV: influenza D virus.

Out of the 2862 pigs sampled, 785 (27.4%) tested positive for at least one pathogen. The most commonly detected pathogen was IAV which was detected in 498 (17.4%) pigs. Influenza A virus was detected in more pigs than the next two most prevalent pathogens combined, PHEV and PPIV1, which were detected in 251 (8.8%) and 201 (7.0%) pigs, respectively. Influenza D virus was the least prevalent and was only detected in two (0.07%) pigs (Table 2). Co-infections were common with 165 (5.8%) pigs testing positive for more than one pathogen, including four (0.14%) pigs testing positive for three pathogens, which were the most pathogens found in co-infected, individual pigs.

**Table 2:** **Respiratory viruses detected in individual pigs at agricultural exhibitions**

Pathogens detected in pigs at exhibitions	Number of pigs positive (%) *n* = 2862
IAV	498 (17.4)
PHEV	251 (8.8)
PPIV1	201 (7.0)
PRRSv	20 (0.7)
IDV	2 (0.07)

Individual swine nasal samples from 102 agricultural exhibitions were tested for five respiratory viruses by real-time reverse transcription polymerase chain reaction. The number of individual pigs positive for each pathogen are shown. Seven hundred eighty-five pigs tested positive for at least one pathogen. Of those, 165 pigs tested positive for > 1 pathogen.

IAV: influenza A virus, PPIV1: porcine parainfluenza virus 1, PHEV: porcine hemagglutinating encephalomyelitis, PRRSv: porcine reproductive and respiratory syndrome virus, IDV: influenza D virus.

## Discussion

Due to widespread movement and prolonged intermingling, exhibition swine and the variety of pathogens they carry should be considered significant for both swine and human health. There has been increased attention on IAV in swine at exhibitions from veterinarians, health officials, and exhibition attendees due to recent IAV zoonotic transmission events [9, 21, 22]. Previous active surveillance has described the epidemiology of IAV in swine at exhibitions in the U.S.; at exhibitions where ≥ 1 pig tests positive for IAV, over 60% of the pigs will have active IAV infection by the conclusion of the exhibition [3]. There were 18 cases of variant influenza infections associated with the IAVs detected in this population of exhibition swine, and IAV should remain a priority for infectious disease surveillance and mitigation in exhibition swine [22]. Several mitigation strategies have been suggested and implemented at exhibitions as the concern for animal and public health rises. Many of these strategies are aimed at reducing the risk of IAV transmission between pigs and humans before, during, and after agricultural swine exhibitions [1, 2]. Still, IAV continues to impact exhibition swine and was detected at almost double the prevalence of PHEV, the next most prevalent virus of those included in this study.

Despite the persistent spread of IAV through exhibition swine and its implications for both animal and human health, other infectious pathogens can be detected in exhibition swine and should not be ignored. Identification of active infections in exhibition swine is vital to protecting animal and human health during agricultural exhibitions. Pigs at exhibitions with at least one pathogen detected in the pigs were more likely to exhibit ILI. However, subclinical infections are common and there is overlap in clinical signs associated with various respiratory pathogens in swine; because of this, clinical signs should not be solely relied upon for the detection and subsequent diagnosis of infectious pathogens in swine at exhibitions [3, 6]. Proper biosecurity should be used at all times, even in the absence of clinical signs, in order to reduce the risk of intra- and interspecies transmission of all pathogens during exhibitions.

Focus on animal and public health at swine exhibitions is a priority, but the potential for introduction of infectious pathogens from exhibition swine into commercial swine facilities should also be considered. An estimated 39% of exhibition pigs are raised near commercial swine, with many of those returning home after attending an exhibition [12]. Detection of pathogens such as PHEV and PRRSv in exhibition swine raises concern for commercial swine due to the potential economic implications should they enter the production system. PRRSv alone causes an estimated $600 million in economic loses annually in the United States swine production system [23]. Detection of PPIV1 and IDV also raises concern, though they are more recently described in swine and their overall consequences to swine health are still unknown [15, 16].

In addition to the five pathogens assessed in the present study, there is potential for other pathogens to travel with and spread through exhibition swine that may also present a threat to commercial swine, including foreign animal-diseases. Exhibition swine should not be disregarded as potential hosts and disseminators of diseases such as African swine fever virus (ASFV); movement and comingling of pigs have been recognized as risk factors for the spread of ASFV in endemic regions [24]. If overlooked, exhibition pigs may create a niche for devastating pathogens, with extensive animal movement resulting in rapid spread across the country. The findings of this study highlight the potential role of exhibition swine in the disease ecology and epidemiology of the United States swine population. Lack of sustained disease surveillance and application of mitigation strategies for swine at agricultural exhibitions will allow for continued variant IAV infections that pose considerable risk to public health and could ultimately lead to a devastating disease epizootic within the U.S. swine herd.

## Data Availability

All data generated or analyzed during this study are included in this published article.

## Abbreviations

IAV: influenza A virus
PHEV: porcine hemagglutinating encephalomyelitis virus
PPIV1: porcine parainfluenza virus 1
PRRSv: porcine reproductive and respiratory syndrome virus
IDV: influenza D virus
rRT-PCR: real-time reverse transcription polymerase chain reaction
ILI: influenza like illness
